# Impact of cellulose and lignin on restoration of vegetation and soil chemical properties for saline-alkali soil of songnen plain

**DOI:** 10.1371/journal.pone.0296366

**Published:** 2024-01-02

**Authors:** Fengzhen Fu, Jianping Luo, Longfei Zhao, Fengjun Yang, Ning Wang

**Affiliations:** 1 College of Horticulture and Landscape Architecture, Heilongjiang Bayi Agricultural University, Daqing, Heilongjiang, China; 2 College of Agronomy, Heilongjiang Bayi Agricultural University, Daqing, Heilongjiang, China; 3 Key Laboratory of Low-Carbon Green Agriculture in Northeastern China, Ministry of Agriculture and Rural Affairs P. R. China, Daqing, Heilongjiang, China; Government College University Lahore, PAKISTAN

## Abstract

To explore the effects of cellulose and lignin on stimulating vegetation restoration and improving soil chemical properties in saline-alkali soil, a large area test was carried out, and 2 treatments were set up: T (cellulose and lignin+ Planted seeds) and CK (Planted seeds). In this study, the species, quantity, plant height, above-ground biomass, biodiversity of vegetation in the treated plots, the determination of soil chemical nutrient content, and the effect of cellulose and lignin on vegetation restoration in saline-alkali land were investigated. The results showed that: 1) Cellulose and lignin contributed to vegetation growth. Compared with CK treatment, plant height and aboveground biomass of T increased by 158.73% and 240.13%, respectively; 2) Cellulose and lignin improved soil structure, and soil porosity, and decreased soil compaction (21.95%); 3) Compared with CK treatment, T treatment decreased soil pH by 0.5 units, total salt content decreased by 30.95%, exchangeable Na^+^ decreased by 63.00%, and exchangeable sodium percentage (*ESP*) decreased by 61.51%. Furthermore, cellulose and lignin effectively improved the physical and chemical properties of saline-alkali soil, promoted the recovery of ecological environment in saline-alkali soil, and improved regional biodiversity, which will provide new methods for soil remediation and improvement in saline-alkali areas.

## Introduction

China has the third largest saline-alkali soil area in the world with a total area of about 1×10^8^ hm^2^. Daqing, a city being located on the Songnen Plain in China, the main saline-alkalization soil distribution area, has a total area of about 1.07×10^5^ hm^2^ of saline-alkalization soil. Furthermore, the low topography and the intense evaporation of the region have intensified the formation of secondary saline-alkalization of the soil [1], not only resulting in a fragile ecological environment, but also preventing the absorption and utilization of nutrients by plant roots due to the poor nutrients and poor physicochemical properties of the soil; meanwhile, the relatively higher pH and salt content have further resulted in low regional biodiversity and severely restricted the development of agriculture and animal husbandry [2–4].

The soil seed bank refers to the sum of litter on soil surface and all active seeds in the soil layer, which constitutes an important material basis for the renewal and restoration of the entire vegetation population [5]. Due to the impact of unfavorable habitats, such as drought, salinity [6–8] and low temperature, the germination of plant seeds in the soil was inhibited. Through reasonably improving the soil surface environment through artificial measures, the germplasm resources in soil can be mobilized, which is of great importance for the restoration of degraded ecosystems as well as vegetation regeneration.

Currently, the major improvement measures for saline-alkalization soil included physical [9–11], chemical [12], hydraulic [13] and biological improvement [14, 15], which were costly [16, 17], prone to secondary pollution, and subject to the influence of external climate [18]. Artificial biomass preparation is based on plant lignin and cellulose as functional biopolymers, and it is mixed with top soil/sand artificially, thereby making it widely applied in recent years to restore vegetation in sandy areas, loess, and roadside slopes due to its functions, including increasing regional vegetation diversity, activating soil seed bank, promoting plant seed germination, and improving micro-ecological environment [18–20]. Compared with biological and chemical crusts, artificial biomass preparations feature the advantages of fast crusting, wide range of applications, independence from extreme natural factors, environmental friendliness, no post maintenance, low cost, *etc*. [21].

Presently, cellulose and lignin were mostly concentrated on the restoration of vegetation on sandy and loess roadside slopes, and there were few reports on the cellulose and lignin promoting the restoration of saline vegetation and improving the chemical properties of saline-alkalization soil. Based on the previous studies [20], this study aims to explore the impact of cellulose and lignin on the restoration of saline-alkalization soil and soil chemical properties with the saline-alkalization area in Daqing City, which will provide new approaches and methods for soil improvement and vegetation restoration in saline-alkalization areas.

## Materials and methods

### Overview of the study area

The experimental area was located in Daqing high tech zone farm (46°36′N,12°59′E), Heilongjiang Province, geographically features the climate type of north temperate sub-European continental monsoon climate, with rain and heat during the same period. The annual average wind velocity is 3.8 m·s^-1^, the annual average precipitation is 428 mm, and the annual average evaporation is 1635 mm. The soil type is alkaline meadow soil with clayey texture and poor permeability; the salinity is dominated by Na_2_CO_3_ and NaHCO_3_, and the vegetational type is *Leymus Chinensis* (Trin. Ex-Bunge) Tzvelev community. Basic chemical properties of soil in the experimental field were shown in Table 1.

**Table 1:** Basic chemical properties of soil in the experimental field.

Soil depth /cm	pH	Organic matter /(g·kg^-1^)	Available phosphorus /(mg·kg^-1^)	Available potassium /(mg·kg^-1^)	Alkaline nitrogen /(mg·kg^-1^)	Soluble salt /(%)	Exchangeable Na^+^ /(cmol·kg^-1^)	Cation exchange capacity /(cmol·kg^-1^)	Exchangeable sodium percentage /(%)
0~5	9.51	14.1	11.57	71.67	18.20	0.56	2.76	14.41	19.15

### Experiment design

The experiment consisted of two treatments, namely T (cellulose and lignin + planted seeds) and CK (planted seeds), and three replications were carried out for treatments. To better avoid the survey error caused by edge effects, the sample plots in this experiment were designed as a circular shape with a plot radius of 5.65 m.

### Preparation of materials and implementation

Materials include lignin (calcium lignosulfonate, CLS, C_20_H_24_CaO_10_S_2_, molecular weight 528), cellulose (hydroxypropyl methyl cellulose, HPMC, C_18_H_38_O_14_, molecular weight 478) (Shandong Heda *Co*., *Ltd*. China), river sand and the compound fertilizer (N15%-P_2_O_5_15%-K_2_O15%, Stanley Agriculture Group *Co*., *Ltd*. China). The plant seeds include *Cosmos bipinnatus* Cav. and *Poa annua* L. (Beijing Forestry University Forest Science *Co*., *Ltd*. China).

On April 7, 2021, the experiment site was subdivided into plots and manually tilled to break the crusts and loosen the soil surface layer to create an excellent habitat for seed germination. The experiment was performed on April 9, and the steps for implementing the T treatment were as follows:

An amount of compound fertilizer was added to the mixture in the ratio of river sand: cellulose (V/V) = 3:1 and then spread evenly over the surface of the treated plots. The amount of cellulose is 10 g·m^-2^, and the amount of compound fertilizer is 4.5 g·m^-2^. Thereafter, plant seeds (*Cosmos bipinnata* Cav. and *Poa annua* L., with an amount of 0.41 g·m^-2^ each species) were sown manually and evenly, and finally, lignin (dosage 150 g·m^-2^) was dissolved in water (dosage 2 L·m^-2^) and splashed manually on the surface of the plots. The implementation of CK and T was the same, and the cellulose and lignin were not included in the materials of CK [20].

### Data acquisition and analysis

On June 10, 2021, at the early stage of vegetation growth, 3 circular quadrats with an area of 1 m^2^ were randomly selected in each plot, and the species, number, plant height and cover of each vegetation in the sample squares were investigated, and the soil compaction was measured at a depth of 0 and 5 cm with a soil compactness meter (SC-900, *Spectrum*, USA). On August 17, 2021, at the peak stage of vegetation growth, the species, number, plant height, cover, and biomass above ground (by flush mowing method, that using scissors to cut plants from the surface) were again measured in the different treatment plots and samples were brought back for laboratory and dried to constant weight. Three 0 and 5 cm soil samples were randomly collected from each plot, dried naturally, sieved, and measured for pH, organic matter, available phosphorus, available potassium, alkaline nitrogen, soluble salts, exchangeable Na^+^ and cation exchange capacity, with the *Methods from Agricultural Chemical Analysis of Soil*, edited by Lu Rukun [22].


$$Exchangeable\;Sodium\;Percentage(ESP)=(exchangeable\;Na^{+}/cation\;exchange\;capacity)\times 100\%$$ (1)

Desalinationrate(%)=(solublesaltinsoilbeforetreatment‐solublesaltinsoilaftertreatment)/solublesaltinsoilbeforetreatment×100% (2)

We calculated the Richness (R), Margalef Index (d_M_), Simpson Index (D), Shannon-Wiener Index (H′), and Pielou Index (J), the calculation formula is as follows:

R=S (3)

$$d_{M}=\left(S-1\right)/lnN$$ (4)

$$D=1-\sum_{i=1}^{s}P_{i}^{2}$$ (5)

$$H\prime =-\sum_{i=1}^{s}P_{i}lnP_{i}$$ (6)

$$J=\frac{H}{lnS}=\left(-\sum_{i=1}^{s}P_{i}lnP_{i}\right)/lnS$$ (7)

In the formula, S represents the number of species in the quadrat; N represents the total number of individuals observed in the quadrat; $P_{i}=\frac{N_{i}}{N}$, *P*_*i*_ represents the ratio of the number of individuals belonging to species *i* to the total number *N*.

Variance analysis(α = 0.05) and *Pearson*’s correlation analysis were performed using SPSS 21.0 (*IBM*, USA); Drawing with Origin 2018 (*Origin Lab*, USA).

### Ethical and plant guidelines statements

Plant seeds, being used in this study, were kindly provided by Pro. Wang Hongyi (College of Horticulture and Landscape Architecture, Heilongjiang Bayi Agricultural University, Daqing, China). Experimental research or field studies on plants, comply with the IUCN Policy Statement on Research Involving Species at Risk of Extinction and the Convention on the Trade in Endangered Species of Wild Fauna and Flora.

## Results

### Impact of cellulose and lignin on plant height and aboveground biomass

**Fig 1:**
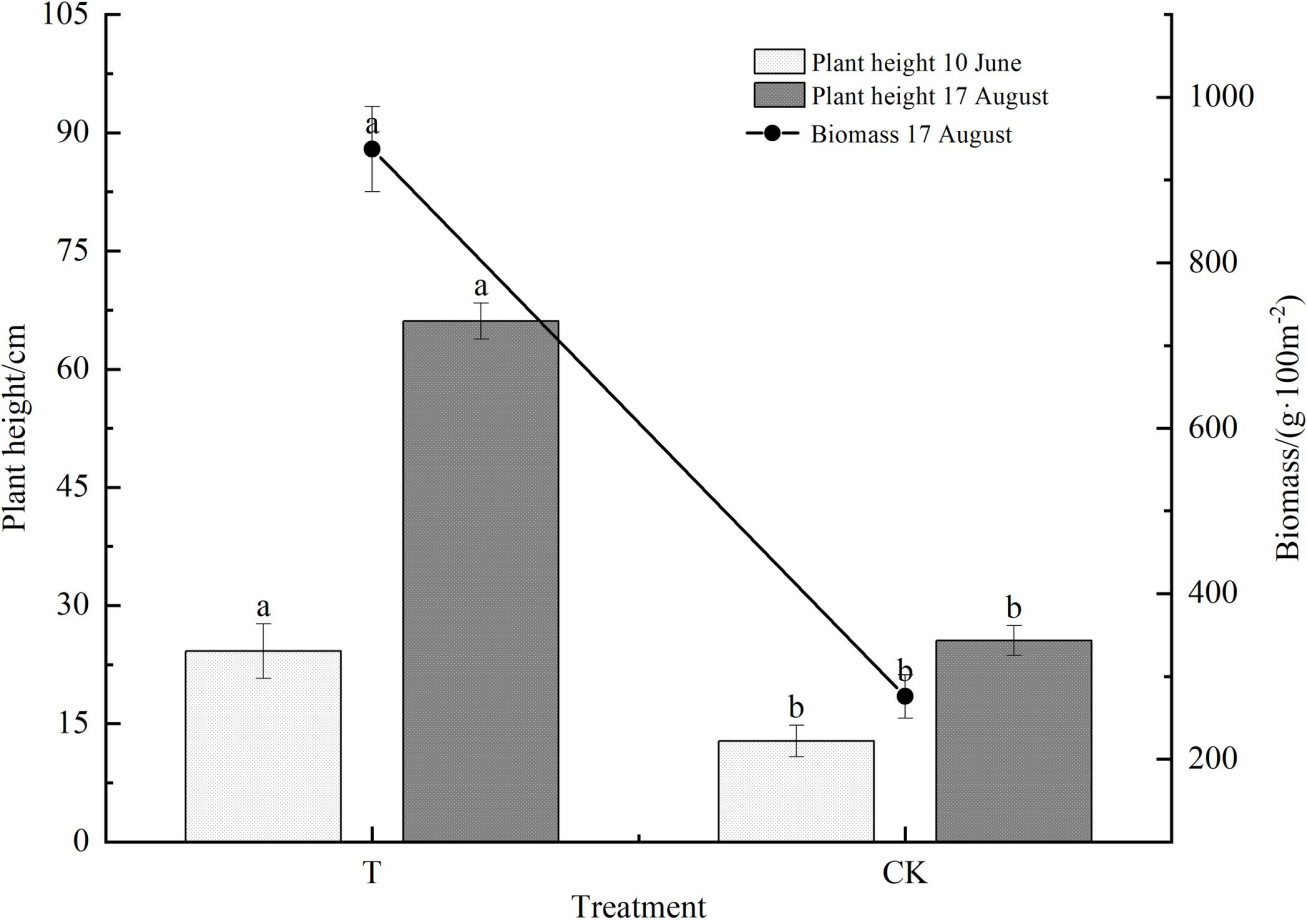
Differences of plant height and biomass under different treatments. Different lowercase letters in the same column indicate significant differences (*p* <0.05) among different treatments.

As shown in Fig 1, there were significant differences in terms of plant height and above-ground biomass at different growth periods for different treatments (*p*<0.05). At the early stage of vegetation growth, the plant height of T treatment was significantly higher than that of CK (*p*<0.05). At the peak stage of vegetation growth, the plant height of T treatment was also remarkedly higher than that of CK (*p*<0.05), with an increase of 158.73%. Moreover, the aboveground biomass of the vegetation in T treatment was also significantly higher than that of CK, which was an increase of 240.13% in comparison to CK. It was observed that cellulose and lignin contributed to the increase in plant height and aboveground biomass.

### Impact of cellulose and lignin on vegetation diversity

Statistical analysis revealed (Table 2) that the vegetation cover, richness, richness index, biodiversity index and evenness index under T treatment were obviously higher than those under CK. At the early stage of vegetation growth, the vegetation cover of T treatment was 62%, an increase of 100% compared to CK, and the richness and each diversity index were greater than those of CK. At the peak stage of vegetation growth, the vegetation cover reached 95% and the number of species was 21 under the T treatment, which was an increase of 90% and 162% respectively, compared with the CK which was an increase of 53.23% in cover and 320% in the number of species respectively, compared with the previous period.

**Table 2:** Vegetation coverage and species diversity indexes of different treatments.

Data	Treatments	Cover degree /%	*Richness*	*Margalef* index	*Simpson* index	*Shannon-wiener* index	*Pielou* index
10/6	T	62a	5a	0.80a	0.64a	1.18a	0.7a
	CK	31b	4a	0.68b	0.32b	0.62b	0.43b
17/8	T	95a	21a	1.85a	0.91a	1.74a	0.93a
	CK	50b	8b	1.27b	0.65b	1.15b	0.72b

Different lowercase letters in the same column indicate significant differences (*p* <0.05) among different treatments.

In Table 3, in addition to the seeds of two plant species (*Cosmos bipinnata* Cav. and *Poa annua* L.) which were sown manually, the seeds of *Lepidium apetalum* Willd., *Astragalus sinicus* L., *Gelsemium elegans* Jussieu., *Polygonum bungeanum* Turcz., *Cirsium setosum*, *Chenopodium album* L., *Cirsium vlassovianum* Fisch. ex DC. and *Pinellia ternata* (Thunb.) Breit. were mobilized from the soil seed bank, involving a total of 21 species in 18 genera from 10 families, in which 19 non-sown species were present in the T-treatment plots. The vegetation survey also showed that: the number of artificially *Cosmos bipinnata* Cav. and *Poa annua* L. in the plots of T treatment accounted for a higher percentage than that in the CK, and the number of other native species also revealed an increased percentage, indicating that a cluster of Cosmo*s bipinnata* -*L*. *chinsis* and ASS. Cosmos *bipinnata*—*Equisetum arvense—L*. *chinsis* had been formed in the T plots, with an obvious trend of positive community succession, while the vegetation in the CK plots was sporadically distributed and had poor growth, and the soil exhibited a trend of sanding or even obvious sanding in some plots. These findings revealed that cellulose and lignin treatment could effectively improve the soil surface ecology, mobilize the soil seed bank, and promote seed germination, which was conducive to the construction and restoration of the ecological environment.

**Table 3:** Species name and taxonomic status of plants under different treatments.

Species name	Taxonomic status	Treatments	
		T	CK
*Poa annua* L.	Poaceae *Poa pratensis*	+	+
*Cosmos bipinnata* Cav.	Compositae *Cosmos* Cav.	+	+
*Potentilla chinensis* Ser.	Rosaceae *Potentilla* L.	+	+
*Plantago depressa* Willd.	Plnataginaceae *Plantago*	+	+
*Setaria viridis* (L.) Beauv.	Graminae *Setaria* Beauv.	+	+
*Polygonum aviculare* L.	Polygonaceae *Polygonum*	+	-
*Artemisia argyi* Levl. et Van	Compositae *Artemisia* L.	+	-
*Pterocypsela elata* (Hemsl.) Shih	Compositae *Lactuca indica* L.	+	-
Miscanthus	Graminae *Miscanthus*	+	-
*Leymus chinensis* (Trin.) Tzvel.	Graminae *Leymus*	+	-
*Lepidium apetalum* Willd.	Brassicaceae *Lepidium* L.	+	-
*Astragalus sinicus* L.	Compositae *Carduus*	+	-
*Gelsemium elegans* Jussieu.	Loganiaceae *Gelsemium* Juss.	+	-
*Polygonum bungeanum* Turcz.	Polygonaceae *Polygonum*	+	-
*Cirsium setosum*	Compositae *Cirsium* Mill. emend. Scop.	+	-
*Chenopodium album* L.	Chenopodiaceae *Chenopodium*.	+	-
*Cirsium vlassovianum* Fisch. ex DC.	Compositae *Cirsium* Mill. emend. Scop.	+	-
*Pinellia ternata* (Thunb.) Breit.	Araceae *Pinellia* Ten.	+	-
*Eleusine indica* (L.) Gaertn.	Graminae *Eleusine*	+	+
*Imperata cylindrica* (L.) Beauv.	Graminae *Imperata*	+	-
*Scirpus triqueter* L.	Cyperaceae *Scirpus*	+	-
*Conyza canadensis* (L.) Cronq.	Compositae *Conyza* Less.	-	+
*Melilotus officinalis* (L.) Pall.	Leguminosae *Osmanthus* Lour.	-	+

+representative species present,—representative species not present.

### Impact of cellulose and lignin on soil compaction

**Fig 2:**
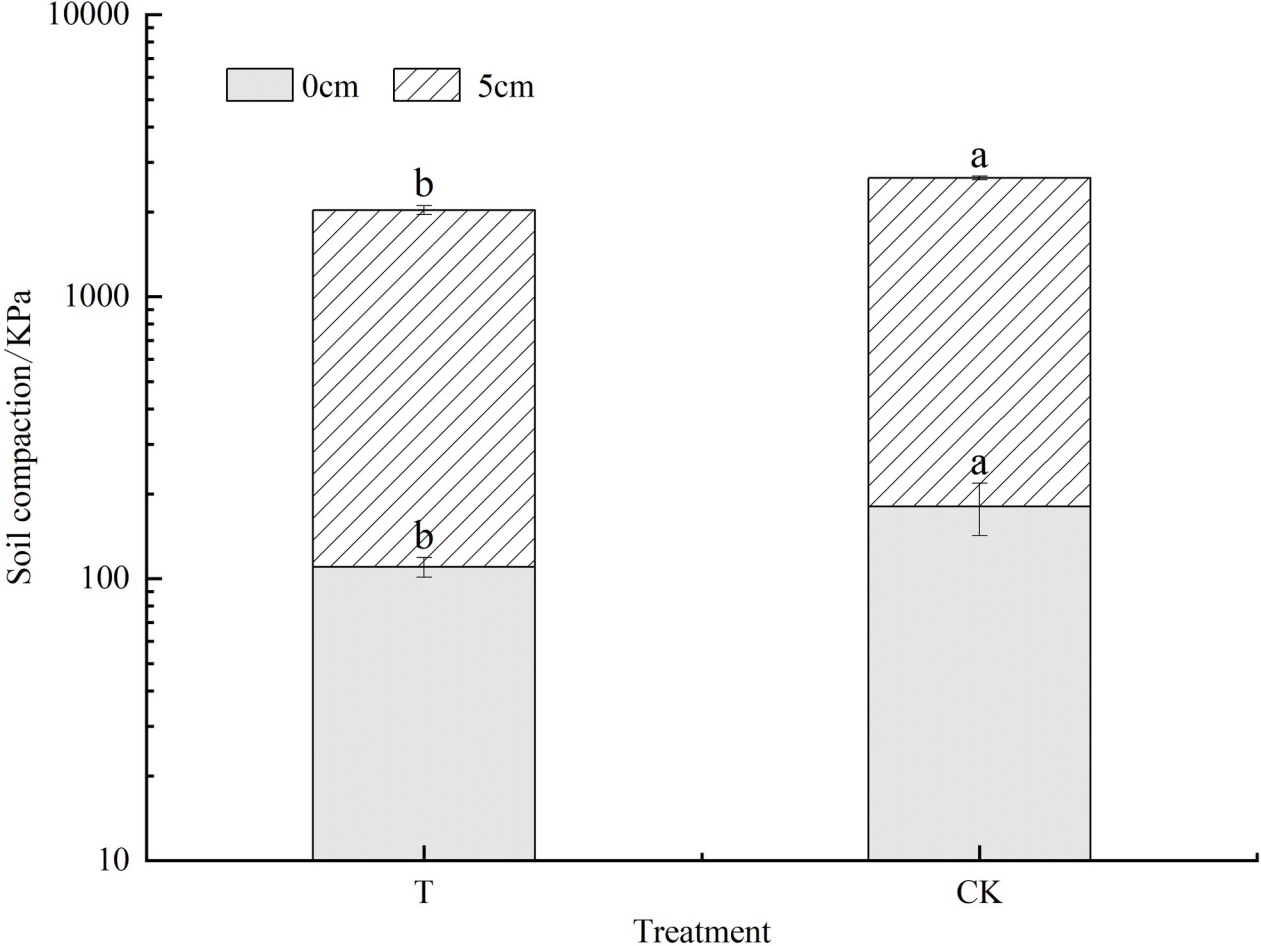
Difference of soil compactness at 5 cm under different treatments. Different lowercase letters in the same column indicate significant differences (*p* <0.05) among different treatments.

Fig 2 showed that, at the soil depth of 0 and 5 cm, there were significant differences in soil compactness between T treatment and CK. The compactness in soil depth of 0 and 5 cm in CK were180±37.75 kPa and 2457±37.82 kPa, respectively, which were remarkably higher than those of T treatment, implying that the applications of cellulose and lignin could improve the soil structure, increase the porosity and looseness of the soil and contribute to the vegetation growth, therefore the vegetation growth would further improve the physicochemical properties of the soil.

### Impact of cellulose and lignin on soil chemical properties

The soil pH, soluble salt and exchangeable sodium percentage of T treatment was significantly lower than those of CK (*p*<0.05) (Table 4). The soil pH of T treatment was 8.71, a decrease of 0.5 units compared to CK; the desalination rate of T treatment reached 48.21%, showing a remarkable desalination effect(*p*<0.05). Furthermore, compared with CK, T treatment could effectively reduce the soil alkalinity by 61.51%, and the content of soil exchangeable Na^+^ in T treatment was decreased by 63.00% compared to CK (*p*<0.05). The soil organic matter, available potassium, available phosphorus and alkaline nitrogen of T treatment were 37.17 g·kg^-1^, 285.75 mg·kg^-1^, 19.53 mg·kg^-1^, 73.27 mg·kg^-1^, respectively, which were an increase of 138.58%, 170.75%, 90.91%, 255.51% compared to CK, respectively.

**Table 4:** Effects of cellulose and lignin on soil chemical properties.

Treatments	pH	Organic matter /(g·kg^-1^)	Available phosphorus /(mg·kg^-1^)	Available potassium /(mg·kg^-1^)	Alkaline nitrogen /(mg·kg^-1^)	Soluble salt /(%)	Exchangeable Na^+^ /(cmol·kg^-1^)	Cation exchange capacity /(cmol·kg^-1^)	Exchangeable sodium percentage /(%)
T	8.71±0.07b	37.17±0.80a	19.53±0.27a	285.75±42.07a	73.27±2.87a	0.29±0.02b	0.84±0.04b	13.51±0.77a	0.06±0.00b
CK	9.20±0.20a	15.58±0.45b	10.23±0.50b	105.54±23.07b	20.61±3.13b	0.42±0.02a	2.27±0.11a	14.05±0.51a	0.16±0.01a

Different lowercase letters in the same column indicate significant differences (*p* <0.05) among different treatments.

### Correlation analysis

#### Correlation analysis of biodiversity, vegetation growth indicators and soil chemical properties

In Table 5, *Pearson’s correlation analysis* of biodiversity, cation exchange capacity, vegetation growth indicators and soil chemical properties in different treatment plots revealed that there were some significant correlations among biodiversity, vegetation growth indicators and soil chemical properties, except for *J* and soil cation exchange. There were significant correlations between vegetation biomass and soil organic matter, or available potassium, alkaline nitrogen, soluble salts, exchangeable Na^+^ and exchangeable sodium percentage. There was a highly significant positive correlation between vegetation biomass and available phosphorus, and a highly significant negative correlation with percentage of soluble salts and exchangeable sodium. Meanwhile, there was a remarkable positive correlation between plant height and soil pH or available potassium, and there was a highly significant positive correlation between plant height and alkaline nitrogen, being a significant negative correlation between plant height and exchangeable Na^+^. In terms of biodiversity, the *D and H′* were only significantly and positively correlated with effective soil phosphorus, but not significantly with other soil chemical properties. *d*_*M*_ and vegetation cover were significantly and positively correlated with soil pH, organic matter, available potassium, or alkaline nitrogen as well as negatively correlated with soil exchangeable Na^+^, where *d*_*M*_ was remarkably and positively correlated with available potassium or alkaline nitrogen, and the vegetation cover was highly and positively correlated with alkaline nitrogen. To improve and restore saline vegetation and increase biodiversity, organic fertilizers and nitrogen, phosphorus and potassium fertilizers have been added to the soil, while, the content of exchangeable Na^+^ and soluble salts in the soil has been controlled.

**Table 5:** Correlation between species diversity indexes、growth traits and soil chemical properties.

	pH	Organic matter	Available phosphorus	Available potassium	Alkaline nitrogen	Soluble salt	Exchangeable Na^+^	Cation exchange capacity	Exchangeable sodium percentage
Margalef index	-0.988*	0.970*	0.901	0.993**	0.992**	-0.877	-0.981*	-0.377	-0.911
Simpson index	-0.801	0.889	0.957*	0.897	0.923	-0.923	-0.910	-0.014	-0.906
Shannon-wiener index	-0.791	0.894	0.967*	0.893	0.913	-0.939	-0.910	0.038	-0.919
Pielou index	-0.512	0.681	0.843	0.667	0.703	-0.813	-0.701	0.307	-0.758
Richness	-0.912	0.978*	0.990**	0.975*	0.973*	-0.974*	-0.985*	-0.083	-0.977*
Cover degree	-0.954*	0.967*	0.944	0.988*	0.999**	-0.913	-0.983*	-0.273	-0.930
Plant height	-0.970*	0.944	0.891	0.980*	0.995**	-0.854	-0.963*	-0.402	-0.883
Vegetation biomass	-0.911	0.990*	0.991**	0.969*	0.948	-0.990**	-0.986*	-0.024	-0.998**

* Indicate significant correlated (*p*<0.05) between groups

**shown great significant correlated (*p*<0.01) between groups.

#### Correlation analysis of soil chemical properties

Table 6 revealed that soil pH and soluble salt content had obvious effects on the contents of soil organic matter, available potassium, available phosphorus, and alkaline nitrogen in different treatment plots. Particularly, soil pH was significantly and negatively correlated with soil organic matter or available potassium or alkaline nitrogen, being significantly and positively correlated with exchangeable Na^+^. In addition, the soil soluble salt was significantly and negatively correlated with organic matter or available phosphorus, and was highly and positively correlated with exchangeable Na^+^ or alkalinity. The soil exchangeable Na^+^ was significantly and negatively correlated with organic matter or available potassium, and had some significant relationships with other components as well. Therefore, the purpose of saline land improvement is to effectively reduce the soil pH and soluble salt content in the soil, and to improve the ability of vegetation to absorb and utilize other effective nutrients.

**Table 6:** Correlations between soil chemical properties.

	pH	Organic matter	Available phosphorus	Available potassium	Alkaline nitrogen	Soluble salt	Exchangeable Na^+^	Cation exchange capacity	Exchangeable sodium percentage
pH	1.00								
Organic matter	-0.959*	1.00							
Available phosphorus	-0.861	0.964*	1.00						
Available potassium	-0.980*	0.991**	0.943	1.00					
Alkaline nitrogen	-0.968*	0.969*	0.931	0.992**	1.00				
Soluble salt	0.844	-0.961*	-0.994**	-0.926	-0.902	1.00			
Exchangeable Na^+^	0.964*	-0.997**	-0.965*	-0.997**	-0.983*	0.954*	1.00		
Cation exchange capacity	0.425	-0.153	0.054	-0.268	-0.312	-0.115	0.189	1.00	
Exchangeable sodium percentage	0.889	-0.982*	-0.989*	-0.952*	-0.924	0.995**	0.974*	-0.034	1.00

* Indicate significant correlated (*p*<0.05) between groups

**shown great significant correlated (*p*<0.01) between groups.

## Discussion

### The effects of cellulose and lignin on stimulating vegetation restoration

Recently, the measures for improving and restoring saline-alkali land by planting saline-tolerance plants had gradually attracted much attentions. Annual saline plants, as pioneer plants, or group plants, were well adaptable to the environment of saline-alkali land in the process of long-term evolution and natural selection, and had a greater potential value for saline-alkali land improvement [5, 6, 8, 19]. This study aimed to increase the vegetation cover and species diversity within the habitat by promoting the restoration of saline land through cellulose and lignin, which would achieve the purpose of saline-alkali land improvement and management. Several studies [23, 24] have shown that the biomass preparations can increase soil temperature and humidity, and have water retention, soil preservation and heat preservation effects. Therefore, the biomass preparations could also provide a favorable condition for the germination of plant seeds, and the hardness of the formed crust was about 160 kPa on average [25], which did not affect the germination of seeds. In this study, at the early stage of vegetation growth, the vegetation cover of T treatment was only 62%, and the difference in richness was not significant compared with CK, and the possible reason was that the early survey time, the cold and long winter in Heilongjiang Province area, the late start of spring, the deep permafrost layer, the slow recovery of temperature and many plant seeds might not have fully sprouted yet, resulting in the low vegetation richness and cover in the cellulose and lignin treatment area. During the peak vegetation growth period, the vegetation growth condition under the cellulose and lignin treatment had a significant advantage, effectively promoting the increase of plant height and above-ground biomass, and improving vegetation cover and biodiversity.

### The effects of cellulose and lignin on improving soil chemical properties

Saline-alkali stress is a major abiotic stress, limiting plant growth and yield [26, 27]. High pH disrupts ion balance and inhibits Na^+^ excretion, which causes damage to plants [28]. In this study, the cellulose and lignin treatment significantly improved the chemical properties of saline soils, reduced the soil pH, soluble salt, and exchangeable Na^+^ content and the alkalinity of the soil, and enhanced the soil fertility and the soil nutrient content, thereby providing certain nutrient conditions for vegetation growth and seed germination.

The cellulose and lignin are a functional polymer, which can improve the soil environment through its own "film-forming" structure on the soil surface, significantly increasing soil temperature and providing a suitable environment for seed germination. Qiu Chaoxia [29] also reported that cellulosic substances could improve soil non-capillary porosity and contributed to soil bulk weight reduction. The treatment of cellulose and lignin significantly improved the soil nutrients and the contents of soil organic matter, available phosphorus, available potassium as well as alkaline decomposition nitrogen. Furthermore, the cellulose and lignin increased the physical and chemical properties of saline soil and promote the growth of saline-tolerant vegetation. A few secretions from the root system of vegetation during growth could increase the soil enzyme activities and activate the organic material in the soil, hence improving the fertility of the soil [16, 19, 20]. On the other hand, the dead branches and leaves of vegetation enter the soil could return the part of the absorbed nutrients to the soil through microbial activities [30, 31], and cellulose and lignin treatments also helped to increase nutrient effectiveness by decreasing soil pH and soluble salts in the amended soil. The decrease in soil pH and soluble salts mainly depended on the acidic nature of the aqueous solution of calcium lignosulfonate, whose 1% aqueous solution had a pH between 4 and 6, which could neutralize the HCO_3_^-^ and CO_3_^2-^ ions in the soil and reduces the potential on the surface of the soil colloid [27, 28]. Meanwhile, plant roots were also able to secrete organic acids, which played a role in lowering soil pH to a certain extent [6]. The surface cover of the soil could reduce the evaporation of the soil and inhibit the rise of salinity [28]. In this experiment, the rate of desalination of the plots treated with cellulose and lignin was increased. Based on the relatively high vegetation cover, the luxuriant branches and leaves could cover the ground, effectively reducing the rise of soil moisture and inhibiting the upward movement of salts, so the "film-forming" structure of the cellulose and lignin also had the effect of covering the ground. Soil exchangeable Na^+^ is a major salt ion in saline soils, and cellulose and lignin treatment reduced the exchangeable Na^+^ content, which might be due to the replacement of Ca^2+^ in calcium lignosulfonate with Na^+^ on soil colloids, which promoted Na^+^ leaching [32–34]. Fan Qianyu *et al*. [30]reported that soil pH and total salt content could be reduced by planting salinity-tolerant forage rape, however, the difference did not reach a significant level compared with before sowing; The activated coke application reduced the soil pH by 0.4%-4.1% and total salt content by 7.4%-8.2%, improving the physicochemical properties of saline-alkali land [35]; The application of coal-fired flue gas desulfurization waste improved saline-alkali soil, resulting in a decrease of soil pH and total salt content in alkalinity, by 43.96% and 38.09%, respectively [36]. Compared to chemicals amendment, such as activated coke and flue gas desulfurization waste, the cellulose and lignin in this study showed more advantages and significant effects in the reduction of soil soluble salts and alkalinity. Nevertheless, there was further improvement in the reduction of soil pH, which will be achieved later by adding a more acidic amendment.

Furthermore, this study only conducted a one-year remediation experiment on saline soil, and its long-term improvement effect needs to be further investigated. Meanwhile, this experiment only explored the chemical properties of soils, and the effects of cellulose and lignin treatment on the physical properties, biological characteristics and soil enzymes of saline-alkali soil are not yet known, and it is also the direction for further research in the future.

## Conclusions

This paper explored the impact of cellulose and lignin treatment on the restoration of saline-alkali land vegetation and soil chemical properties. The cellulose and lignin promoted plant height and aerial biomass and were effective in improving the ecological environment of the soil surface, promote seed germination, which contributed to the establishment and restoration of the ecological environment. In addition, the cellulose and lignin treatment also improved the structure of saline soil, added porosity and looseness to the soil, and reduced the soil compactness. Those finding implied that the cellulose and lignin could effectively improve the chemical properties of saline-alkali soil.
